# Study of Two Dose Regimens of Ticagrelor Compared With Clopidogrel in Patients Undergoing Percutaneous Coronary Intervention for Stable Coronary Artery Disease

**DOI:** 10.1161/CIRCULATIONAHA.118.034790

**Published:** 2018-09-24

**Authors:** Rachel C. Orme, William A.E. Parker, Mark R. Thomas, Heather M. Judge, Kathleen Baster, Wael Sumaya, Kenneth P. Morgan, Hannah C. McMellon, James D. Richardson, Ever D. Grech, Nigel M. Wheeldon, Ian R. Hall, Javaid Iqbal, David Barmby, Julian P. Gunn, Robert F. Storey

**Affiliations:** 1 Department of Infection, Immunity and Cardiovascular Disease (R.C.O., W.A.E.P., M.R.T., H.M.J., W.S., H.C.M., J.I., J.P.G., R.F.S.), University of Sheffield, United Kingdom.; 2 Statistical Services Unit, (K.B.), University of Sheffield, United Kingdom.; 3 Sheffield Teaching Hospitals National Health Service Foundation Trust, United Kingdom (R.C.O., W.A.E.P., M.R.T., W.S., K.P.M., H.C.M., J.D.R., E.D.G., N.M.W., I.R.H., J.I., D.B., J.P.G., R.F.S).; 4 University of Birmingham, United Kingdom (M.R.T.).; University of Nottingham; Nottingham University Hospitals NHS Trust; Nottingham University Hospitals NHS Trust

**Keywords:** adenosine, blood platelets, clopidogrel, coronary artery disease, percutaneous coronary intervention, ticagrelor

## Abstract

**Background:**

Ticagrelor has superior efficacy to clopidogrel in the management of acute coronary syndromes but has not been assessed in patients undergoing percutaneous coronary intervention for stable coronary artery disease. We compared the pharmacodynamic effects of ticagrelor and clopidogrel in this stable population.

**Methods:**

One hundred eighty aspirin-treated stable coronary artery disease patients, who were planned to undergo elective percutaneous coronary intervention in a single center, were randomized 1:1:1 to either a standard clopidogrel regimen or 1 of 2 regimens of ticagrelor, either 90 mg (T90) or 60 mg twice daily (T60), both with a 180 mg loading dose. Cellular adenosine uptake was assessed, at the time of the procedure and pre- and postdose at 1 month, by adding adenosine 1 µmol/L to aliquots of anticoagulated whole blood and mixing with a stop solution at 0, 15, 30, and 60 seconds, then measuring residual plasma adenosine concentration by high-performance liquid chromatography. Systemic plasma adenosine concentration and platelet reactivity were assessed at the same timepoints. High-sensitivity troponin T was measured pre- and 18 to 24 hours postpercutaneous coronary intervention.

**Results:**

One hundred seventy-four patients underwent an invasive procedure, of whom 162 received percutaneous coronary intervention (mean age 65 years, 18% female, 21% with diabetes mellitus). No effect on in vitro adenosine uptake was seen postdose at 1 month for either ticagrelor dose compared with clopidogrel (residual adenosine at 15 seconds, mean±SD: clopidogrel 0.274±0.101 µmol/L; T90 0.278±0.134 µmol/L; T60 0.288±0.149 µmol/L; *P*=0.37). Similarly, no effect of ticagrelor on in vitro adenosine uptake was seen at other timepoints, nor was plasma adenosine concentration affected (all *P*>0.1). Both maintenance doses of ticagrelor achieved more potent and consistent platelet inhibition than clopidogrel (VerifyNow P2Y_12_ reaction units, 1 month, mean±SD: predose, T60: 62±47, T90: 40±38, clopidogrel 181±44; postdose, T60: 34±30, T90: 24±21, clopidogrel 159±57; all *P*<0.0001 for ticagrelor versus clopidogrel). High platelet reactivity was markedly less with both T60 and T90 compared with clopidogrel (VerifyNow P2Y_12_ reaction units>208, 1 month postdose: 0%, 0%, and 21%, respectively). Median (interquartile range) high-sensitivity troponin T increased 16.9 (6.5–46.9) ng/L for clopidogrel, 22.4 (5.5–53.8) ng/L for T60, and 17.7 (8.1–43.5) ng/L for T90 (*P*=0.95). There was a trend toward less dyspnea with T60 versus T90 (7.1% versus 19.0%; *P*=0.09).

**Conclusions:**

Maintenance therapy with T60 or T90 had no detectable effect on cellular adenosine uptake at 1 month, nor was there any effect on systemic plasma adenosine levels. Both regimens of ticagrelor achieved greater and more consistent platelet inhibition than clopidogrel but did not appear to affect troponin release after percutaneous coronary intervention.

**Clinical Trial Registration:**

URL: https://www.clinicaltrials.gov. Unique identifier: NCT02327624.

## Abstract 2

Supplemental Digital Content is available in the text.

**Clinical Perspective**

**What Is New?**

- Ticagrelor does not significantly impair adenosine uptake or increase circulating adenosine levels in patients with stable coronary artery disease.
- Ticagrelor 60 mg or 90 mg twice daily provides greater and more consistent platelet inhibition than clopidogrel in stable coronary artery disease patients undergoing elective percutaneous coronary intervention.
- More potent platelet P2Y_12_ inhibition did not modify troponin release related to percutaneous coronary intervention.

**What Are the Clinical Implications?**

- Further studies of ticagrelor 60 mg twice daily are warranted in stable coronary artery disease patients undergoing percutaneous coronary intervention.
- Asymptomatic troponin release may not be a suitable end point for assessing the impact of greater platelet inhibition in stable coronary artery disease patients undergoing percutaneous coronary intervention.

**Editorial, see p 1301**

Dual antiplatelet therapy with aspirin and an oral platelet P2Y_12_ receptor antagonist is the standard therapy for patients undergoing percutaneous coronary intervention (PCI). Three oral platelet P2Y_12_ receptor antagonists are currently available: the thienopyridines clopidogrel and prasugrel, and the nonthienopyridine, reversibly binding drug ticagrelor.^1–4^ In the absence of contraindications or concurrent oral anticoagulant therapy, ticagrelor is recommended in preference to clopidogrel for patients with acute coronary syndromes, including those managed with PCI, but it has not been assessed in patients undergoing PCI for stable coronary artery disease (CAD).^1–3^ Similarly, prasugrel is recommended in preference to clopidogrel for acute coronary syndrome patients managed with PCI, but is not licensed for use in stable CAD.^1–3^ Consequently, aspirin and clopidogrel remain the predominant dual antiplatelet therapy strategy in stable CAD patients undergoing PCI.

Thienopyridines, such as clopidogrel, are prodrugs that require hepatic metabolism to generate active metabolites that bind irreversibly to the platelet P2Y_12_ receptor, blocking the binding of ADP to this receptor.^5^ The efficacy of clopidogrel is limited in some individuals due to poor efficiency of active metabolite formation and poor pharmacodynamic response has been associated with increased risk of stent thrombosis in clopidogrel-treated patients.^6,7^

Ticagrelor is not a prodrug but does have an active metabolite, AR-C124910XX, that is equipotent to ticagrelor and contributes approximately 30% of the total inhibitory effect.^5,8,9^ Ticagrelor achieves a consistent high level of platelet P2Y_12_ inhibition following a loading dose (although onset of action can be delayed in patients with ST-elevation myocardial infarction [MI]^10,11^), as well as during maintenance therapy with either 90 mg or 60 mg BID in patients with prior MI.^9^ Ticagrelor and AR-C124910XX also have weak inhibitory effects on cellular adenosine uptake via ENT-1 (equilibrative nucleoside transporter 1), although the clinical significance of this effect remains uncertain.^12–14^ The effects of ticagrelor 60 mg BID on adenosine metabolism have not been previously reported. In the STEEL-PCI study (Study of Two Doses of Ticagrelor in PCI; NCT02327624), we assessed and compared the effects of ticagrelor and clopidogrel on cellular adenosine uptake, as well as platelet reactivity in stable CAD patients undergoing PCI.

## Methods

The data, analytic methods, and study materials will not be made available to other researchers for purposes of reproducing the results or replicating the procedure.

### Study Design

**Figure 1.**
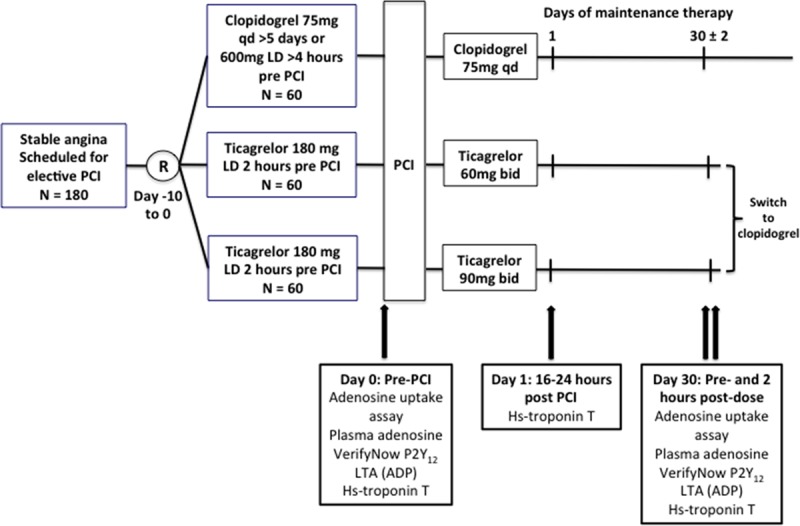
**Study design.** Hs indicates high-sensitivity; LD, loading dose; LTA (ADP), light transmittance aggregometry with ADP; PCI, percutaneous coronary intervention; and R, randomization.

One hundred eighty patients with stable CAD provided written informed consent and were enrolled into the STEEL-PCI study, conducted at a single center (Northern General Hospital, Sheffield, United Kingdom). All patients had had previous coronary angiography and were planned to undergo PCI. Other inclusion as well as exclusion criteria are shown in the online-only Data Supplement. The study was performed according to a protocol approved by the National Research Ethics Service and regulatory authorities. Aspirin-treated patients who provided informed consent were randomized in a 1:1:1 fashion to receive open-label treatment with a 180 mg loading dose of ticagrelor at 2 hours pre-PCI, followed by either 60 mg BID or 90 mg BID for 1 month or a standard loading regimen of clopidogrel (600 mg at least 4 hours prior to procedure or maintenance therapy with 75 mg for at least 5 days), followed by 75 mg QD for 1 month (Figure 1). Blood samples were collected at the time of PCI, either from a large antecubital vein using a 21-G needle and syringe with minimum use of tourniquet or from the arterial sheath, before the administration of heparin. Patients attended the morning after PCI for collection of venous blood samples by venipuncture. At 1 month post-PCI, patients attended before the morning maintenance dose of study medication for further collection of venous blood samples. The maintenance dose was then administered and a further blood sample obtained 2 hours later. Patients were instructed to return unused study medication at their 1-month visit, and compliance was assessed by pill-counting. When indicated, patients were switched to open-label clopidogrel at the 1-month visit by administration of a loading dose 24 hours after the last dose of ticagrelor, as recommended.^15,16^ Staff of the Clinical Research Office of Sheffield Teaching Hospitals National Health Service Foundation Trust monitored the study, and a data monitoring committee periodically reviewed the conduct of the study and clinical outcomes.

### Adenosine Uptake and Plasma Adenosine Level

For adenosine reuptake measurements, blood was collected into a standard ethylenediaminetetraacetic acid tube and then aliquots pipetted into tubes containing adenosine (final concentration: 1 µmol/L). Uptake of adenosine was halted by the addition of a cold pharmacological stop solution (2 parts blood:1 part stop solution) at 0, 15, 30, or 60 seconds after mixing blood with the adenosine. The stop solution was composed of dipyridamole 40 µmol/L, disodium ethylenediaminetetraacetic acid 13.2 mmol/L, erythro-9-(2-hydroxy-3-nonyl)adenine 50 µmol/L, α,β-methylene adenosine 5’-diphosphate 200 µmol/L, iodotubercidin 50 µmol/L, and p-nitrobenzylthioinosine 40 µmol/L in 0.9% wt/vol sodium chloride. Adenosine concentration was measured using high-performance liquid chromatography (see the online-only Data Supplement).

Samples for plasma adenosine concentration measurement were collected into S-Monovette tubes containing the stop solution and immediately placed on ice before centrifugation at 1500*g*. Adenosine concentration was then measured as described above.

### VerifyNow P2Y_12_ Assay

Whole blood was collected into 2-mL Greiner Bio-One citrate tubes and gently mixed before analysis after 20 minutes using the VerifyNow P2Y_12_ assay (Accumetrics Inc, USA). P2Y_12_ reaction units (PRU) and VerifyNow percentage inhibition (estimated using the Base channel result as 100% response) were recorded.

### Light Transmittance Aggregometry

Light transmission aggregometry (LTA) was performed using a PAP8 aggregometer (Biodata; Horsham, USA) with ADP 20 μmol/L as the agonist. Maximum percentage LTA responses were recorded.

### High-Sensitivity Troponin T

High-sensitivity troponin T was determined in serum samples (Elecsys assay, Roche, on a Cobas E602 analyser) before PCI and the morning after PCI.

### Pharmacokinetic Analysis

Plasma derived from blood anticoagulated with lithium heparin was stored at –80°C prior to analysis. Plasma concentrations of ticagrelor and AR-C124910XX were determined using liquid chromatography with tandem mass spectrometry by York Bioanalytical Solutions (Upper Poppleton, United Kingdom).^17^

### Genetic Analysis

DNA was extracted from whole blood and analyzed for relevant genetic variants of *CYP2C19*, *CY3A43*, *UGT2B7*, and *SLC01B1* (see the online-only Data Supplement).

### Sample Size and Statistical Analysis

The primary end point of the study was in vitro adenosine uptake postmaintenance dose at 1 month, measured as residual adenosine concentration at 15 seconds after ex vivo addition of adenosine. The sample size was based on (1) our preliminary in vitro studies of adenosine uptake indicating 15 seconds as the optimal time for assessing residual adenosine concentration, and previous data indicating an estimated mean (±SD) residual adenosine concentration at 15 seconds postmixing in the adenosine uptake assay of 0.80±0.051 µmol/L for the ticagrelor 90 mg group and 0.45±0.068 µmol/L for clopidogrel^18^; and (2) the assumption that the effects of ticagrelor 60 mg would yield levels between those with ticagrelor 90 mg and clopidogrel. Data on 42 patients per group were required in each group to provide >90% power to detect a 0.05 µmol/L higher mean residual adenosine level in the ticagrelor 60 mg group compared with the clopidogrel group, with a significance threshold of 0.05 and assuming a common SD of 0.06 µmol/L, and >99% power to detect a similar difference between the ticagrelor 90 mg and clopidogrel groups to that previously reported. Sixty patients were, therefore, required in each group to allow for 30% dropout or sample failure at 1 month. Secondary endpoints were plasma adenosine concentration, platelet function measurements, and the PCI-induced troponin release (determined as increase from pre-PCI to post-PCI). Based on our previous work,^8,9^ the proposed sample size provided >90% power to detect expected differences in platelet aggregation, assessed by either VerifyNow P2Y_12_ assay or LTA, between ticagrelor and clopidogrel, with a significance threshold of 0.01 (to allow for multiple testing), allowing for 30% dropout or sample failure at 1 month.

Data were analyzed using SAS version 9.3 (SAS Institute, USA) and expressed as mean and SD for normally distributed data or median and interquartile range for nonparametric data. Continuous data were compared using the Kruskal-Wallis test, where appropriate using the Mann-Whitney test for pairwise comparisons, as indicated in Results. Categorical variables were compared using the chi-square test or Fisher exact test, as indicated in Results. High platelet reactivity was defined as VerifyNow PRU >208 or LTA response >59%.^9^ MI was defined according to the Third Universal Definition.^19^ Bleeding events were defined according to the PLATO study (Platelet Inhibition and Patient Outcomes) criteria.^20^

## Results

### Study Population

**Figure 2.**
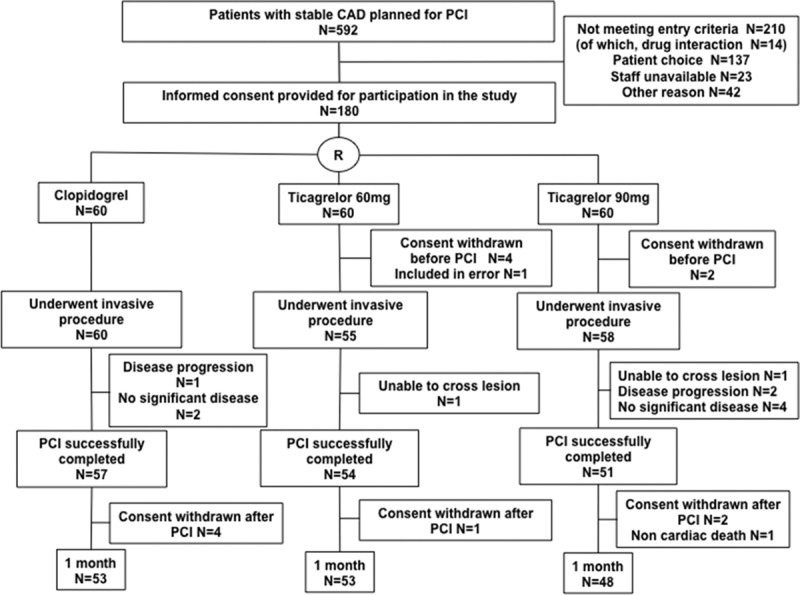
**CONSORT study (Consolidated Standards of Reporting Trials) flow diagram.** Number of patients in each of the 3 treatment groups (clopidogrel, ticagrelor 60 mg BID, and ticagrelor 90 mg BID) at each stage of the study. CAD indicates coronary artery disease; PCI, percutaneous coronary intervention; and R, randomization.

One hundred eighty patients were recruited to the study (Figure 2). Sixty patients in the clopidogrel group, 56 in the ticagrelor 60 mg BID group, and 58 in the ticagrelor 90 mg BID group underwent an invasive procedure. Some patients did not proceed to PCI for several reasons, including significant disease progression requiring surgical management or nonflow-limiting coronary stenosis on updated angiography. One hundred fifty-five patients completed the study period of maintenance therapy with clopidogrel 75 mg QD (n=53), ticagrelor 60 mg BID (n=54), or ticagrelor 90 mg BID (n=48). One patient in the ticagrelor 60 mg BID group was subsequently found to have been taking an excluded medication (a strong CYP3A [cytochrome P450, family 3, subfamily A] inducer), and was excluded from the main analysis; however, their results are included in the online-only Data Supplement. The demographic characteristics, cardiovascular risk factors, and concomitant medications were well matched between the groups at randomization and subsequent timepoints, as were the procedural characteristics for those proceeding with PCI (Table 1 and Tables I and II in the online-only Data Supplement). At the time of their procedure, 100% patients were receiving aspirin 75 mg daily and continued on this for the duration of the study.

**Table 1.**
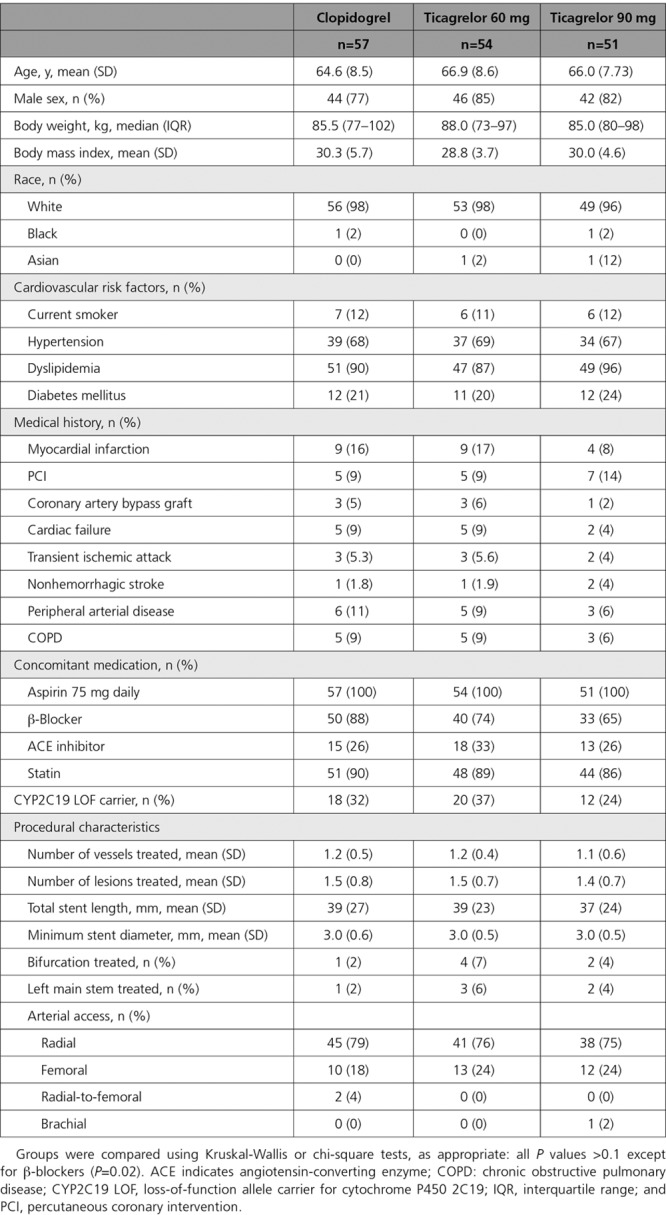
Demographic and Procedural Characteristics and Medications for Patients Proceeding With Percutaneous Coronary Intervention

### Adenosine Uptake and Plasma Adenosine Level

**Figure 3.**
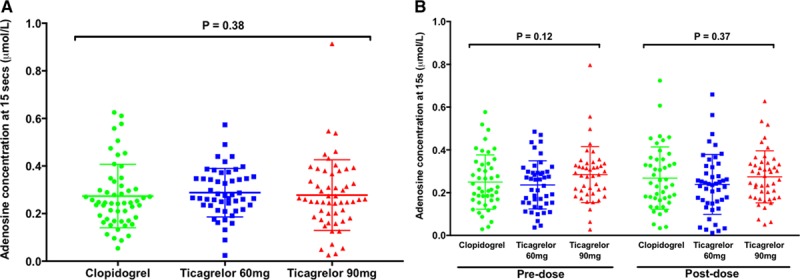
**Whole blood in vitro adenosine uptake.** Residual adenosine levels at 15 seconds after mixing adenosine 1 μmol/L with blood samples obtained (**A**) at the time of percutaneous coronary intervention following a standard loading regimen of clopidogrel (n=54) or 180 mg loading dose of ticagrelor (n=50 and 54 for 60 mg and 90 mg groups, respectively), and (**B**) after 1 month of treatment, premaintenance dose and postmaintenance dose for each of the 3 treatment groups (clopidogrel 75 mg QD: n=45; ticagrelor 60 mg BID: n=46; and ticagrelor 90 mg BID; n=43 and 45). Horizontal bars indicate mean±SD. *P* values determined using 3-group comparison with Kruskal-Wallis test.

**Figure 4.**
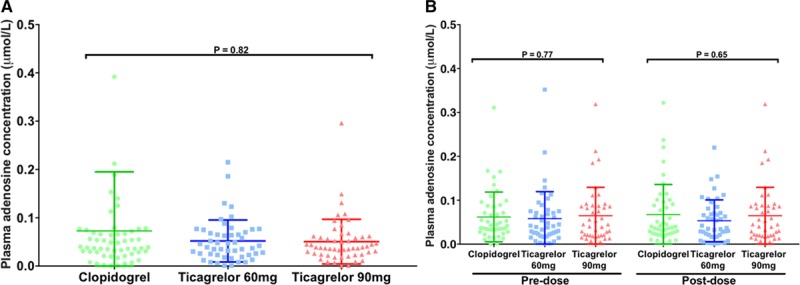
**Plasma adenosine concentration.** Plasma adenosine levels (**A**) at the time of percutaneous coronary intervention following a standard loading regimen of clopidogrel (n=56) or 180 mg loading dose of ticagrelor (n=50 and 54 for 60 mg and 90 mg groups, respectively) and (**B**) after 1 month of treatment, premaintenance dose and postmaintenance dose for each of the 3 treatment groups (clopidogrel 75 mg QD: n=45; ticagrelor 60 mg BID: n=46; and ticagrelor 90 mg BID: n=43). Horizontal bars show mean±SD. *P* values determined using 3-group comparison with Kruskal-Wallis test.

No effect on in vitro adenosine uptake was seen with a ticagrelor loading dose or the 90 mg or 60 mg BID maintenance doses compared to clopidogrel at the time of PCI or at 1 month (Figure 3 and Figure I in the online-only Data Supplement). Similarly, there was no impact of ticagrelor at any time point on plasma adenosine level (Figure 4).

### VerifyNow P2Y_12_ Assay and Light Transmittance Aggregometry

**Figure 5.**
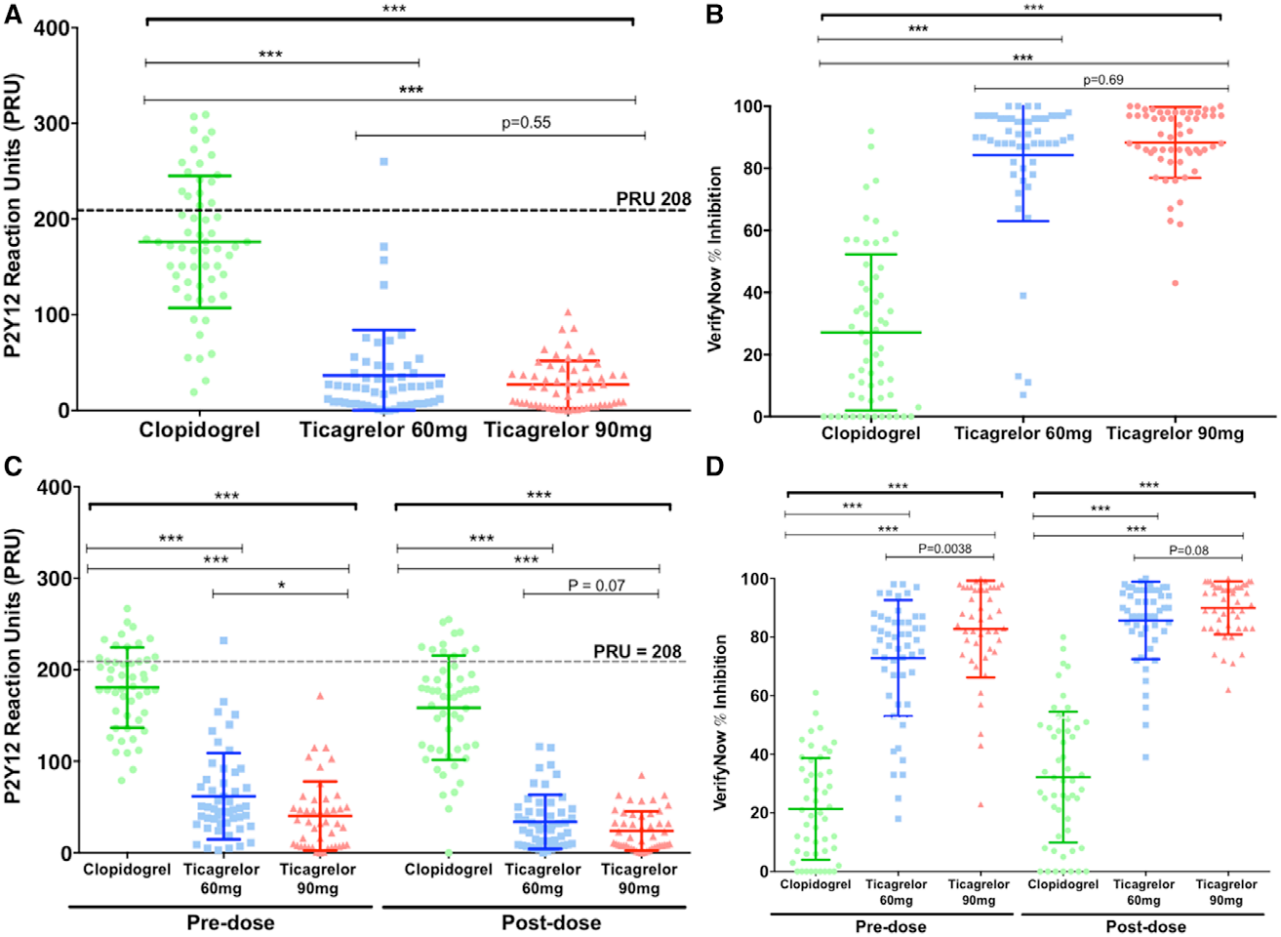
**VerifyNow P2Y_12_ Assay Results.** Individual VerifyNow P2Y_12_ assay results expressed as (**A** and **C**) P2Y_12_ reaction units (PRU) and (**B** and **D**) VerifyNow percentage inhibition, (**A** and **B**) at the time of percutaneous coronary intervention following a standard loading regimen of clopidogrel (n=59) or 180 mg loading dose of ticagrelor (n=54 and 58 for 60 mg and 90 mg groups, respectively) and (**C** and **D**) after 1 month of treatment, premaintenance dose and postmaintenance dose for each of the 3 treatment groups (clopidogrel 75 mg QD: n=52; ticagrelor 60 mg BID: n=52; and ticagrelor 90 mg BID: n=48). The dashed lines indicate a level of 208 PRU as a threshold for high platelet reactivity. Horizontal bars indicate mean±SD. *P* values determined using 3-group comparison with Kruskal-Wallis test with pairwise comparisons using Mann-Whitney test; * *P*<0.01; *** *P*<0.0001.

**Figure 6.**
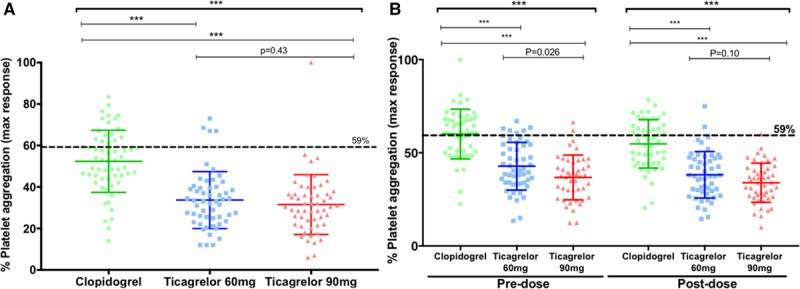
**ADP-induced platelet aggregation determined by LTA.** Individual results for the platelet aggregation measured by light transmittance aggregometry in response to ADP 20 μmol/L (**A**) at the time of percutaneous coronary intervention following a standard loading regimen of clopidogrel (n=59) or 180 mg loading dose of ticagrelor (n=54 and 55 for 60 mg and 90 mg groups, respectively) and (**B**) after 1 month, premaintenance dose and postmaintenance dose for each of the 3 treatment groups (clopidogrel 75 mg QD: n=50 and 51; ticagrelor 60 mg BID: n=51 and 52; and ticagrelor 90 mg BID: n=45 and 48). The dashed lines indicate a level of 59% as a threshold value for high platelet reactivity. Horizontal bars indicate mean±SD. *P* values determined using 3-group comparison with Kruskal-Wallis test with pairwise comparisons using Mann-Whitney test; *** *P*<0.0001. LTA indicates light transmittance aggregometry; and max, maximum.

Ticagrelor 180 mg loading dose achieved greater and more consistent platelet inhibition than clopidogrel at the time of PCI when assessed by the VerifyNow P2Y_12_ assay (Figure 5A and 5B). Both maintenance doses of ticagrelor achieved greater and more consistent platelet inhibition than clopidogrel 75 mg daily at 1 month (Figure 5C and 5D). The mean (±SD) predose PRU values were 62±47 versus 40±38 (*P*<0.01) for the 60 mg versus 90 mg ticagrelor doses, and postdose values were 34±30 versus 24±21 (*P*=0.09), respectively; corresponding PRU values for clopidogrel-treated patients were 181±44 predose and 159±57 postdose (all *P*<0.0001 versus both ticagrelor groups). The mean LTA responses were also significantly lower in the ticagrelor groups compared with the clopidogrel group, both at the time of PCI and at 1 month (Figure 6).

High platelet reactivity, as assessed by the VerifyNow P2Y_12_ assay, was seen infrequently in the ticagrelor group (n=1) at the time of PCI (Table 2). This patient also had high platelet reactivity when assessed by LTA. No patients in the ticagrelor 90 mg BID group had high platelet reactivity (PRU>208) at 1 month compared to 1 patient in the ticagrelor 60 mg BID group. This patient had a PRU value of 232 at 1 month predose and 39 postdose, with a PRU of 1 at the time of PCI; their drug compliance at 1 month was calculated at 100%. High platelet reactivity was more common in the clopidogrel group at all the timepoints compared to both ticagrelor groups (Table 2).

**Table 2.**
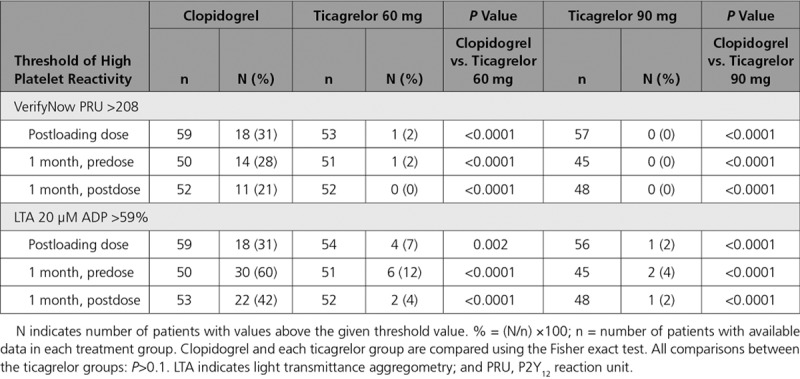
Proportions of Patients With High Platelet Reactivity According to Predefined Threshold Values

There were a small number of patients with high platelet reactivity in the ticagrelor groups (<15%) according to LTA responses compared to greater proportions in the clopidogrel group (>30%) at each timepoint (Figure 6B and Table 2).

### Efficacy, Safety, and Tolerability

There were no MIs, strokes, or cardiac deaths in any of the groups at 30 days. There was only 1 death, which occurred as a result of sepsis following mesenteric infarction that did not appear to be related to the PCI procedure. There was no effect of the higher levels of platelet inhibition with ticagrelor on PCI-induced increase in high-sensitivity troponin T: median (interquartile range) increases the morning after PCI were 16.9 (6.5–46.9) ng/L for the clopidogrel group, 22.4 (5.5–53.8) ng/L for the ticagrelor 60 mg group, and 17.7 (8.1–43.5) ng/L for the ticagrelor 90 mg group (*P*=0.95, Kruskal-Wallis test).

The tolerability of the ticagrelor 60 mg BID dose appeared slightly better than the 90 mg BID dose due to less frequent dyspnea events in the 60 mg group (7.1% versus 19.0%; *P*=0.09) (Table III in the online-only Data Supplement). Two patients (3.6%) in the ticagrelor 60 mg group and 3 patients (5.2%) in the ticagrelor 90 mg group stopped study medication prematurely due to adverse effects. There was no reported dyspnea in the clopidogrel group, and no patients stopped clopidogrel prematurely due to adverse effects. There were no PLATO-defined major or minor bleeds and no major adverse cardiac events or stent thrombosis events in any of the treatment groups.

### Pharmacokinetics

The mean plasma levels of ticagrelor and AR-C124910XX following ticagrelor 180 mg loading dose were 1109±549 and 223±121 ng/mL, respectively (Figure IIA in the online-only Data Supplement). After 1 month maintenance therapy with either ticagrelor 60 mg or ticagrelor 90 mg BID, predose mean levels of ticagrelor were 278±217 and 365±189 ng/mL, respectively, and predose mean levels of AR-C124910XX were 97±55 and 127±73 ng/mL, respectively. Postdose mean levels of ticagrelor were 510±281 and 776±347 ng/mL, and mean levels of AR-C124910XX were 135±69 and 199±96 ng/mL, respectively (Figure IIB in the online-only Data Supplement).

### Genetic Analysis

The ticagrelor loading dose and both ticagrelor maintenance doses achieved greater platelet inhibition than clopidogrel in those who either did or did not carry *CYP2C19* loss-of-function alleles (Tables IV and V in the online-only Data Supplement). The other genetic variants studied did not significantly influence the pharmacodynamic and pharmacokinetic results (Tables VI through XI in the online-only Data Supplement).

## Discussion

In this study, we compared the pharmacodynamic effects of ticagrelor and clopidogrel, obtaining data on the 60 mg BID dose of ticagrelor for the first time in stable CAD patients undergoing PCI and collecting preliminary efficacy, safety, and tolerability data on the 2 doses of ticagrelor in this setting. Consistent with previous comparisons of the ticagrelor 180 mg loading dose and 90 mg twice-daily maintenance dose with standard regimens of clopidogrel in other clinical settings, we confirmed that the ticagrelor loading dose and maintenance doses achieved greater and more consistent levels of platelet inhibition compared to standard regimens of clopidogrel in stable CAD patients at the time of, and 1 month after, PCI. Of note, we show that the ticagrelor 60 mg twice-daily maintenance dose provides much more consistent platelet inhibition than clopidogrel, even in those with normal CYP2C19 activity as predicted by *CYP2C19* genotyping. Our data are broadly consistent with previously reported data on ticagrelor 90 mg and 60 mg twice daily in patients with prior MI.^9,21^ Our finding of significant difference in predose platelet reactivity during maintenance therapy in the 2 ticagrelor groups, in contrast to lack of significance of this comparison in the PEGASUS-TIMI 54 platelet function substudy (Prevention of Cardiovascular Events in Patients With Prior Heart Attack Using Ticagrelor Compared to Placebo on a Background of Aspirin-Thrombolysis in Myocardial Infarction 54),^9^ likely reflects small sample sizes in both studies, limiting the power to detect such a difference. Dyspnea was more frequent in the ticagrelor groups, and this is a well-characterized adverse effect of ticagrelor that is usually mild or moderate in severity, as confirmed here.^22–24^ The lower rates of dyspnea in the ticagrelor 60 mg group, combined with the reliable P2Y_12_ inhibition, as also previously demonstrated in the PEGASUS-TIMI 54 study,^9,24^ favor this dose for further exploration in clinical outcomes studies.

Contrary to some previous published studies,^18,25^ we found no evidence of any effect of the ticagrelor regimens on cellular adenosine uptake or plasma adenosine concentration. The reasons for this are unclear since our data show clearly that the assay assessed adenosine uptake over 1 minute in whole blood samples, with the expected baseline levels of adenosine after in vitro addition of 1 µmol/L (indicating efficacy of the stop solution in preventing further adenosine uptake) and almost complete adenosine uptake at 1 minute (indicating efficacy of the stop solution in preventing adenosine generation). Our stop solution for adenosine metabolism included additional inhibitors to those used by Bonello et al,^18^ including p-nitrobenzylthioinosine as an additional inhibitor of adenosine uptake and iodotubercidin as a potent adenosine kinase inhibitor, and therefore may have been more effective. In agreement with our findings, a recent study in healthy volunteers found no impact of ticagrelor on plasma adenosine level.^26^ Furthermore, using the same methodology, we found no impact of ticagrelor on plasma adenosine concentration in acute coronary syndrome patients awaiting coronary artery bypass graft surgery, suggesting that the nature of the patient population in our current study was not a determinant of the findings.^27^ In vitro studies predict little effect of ticagrelor on adenosine uptake at therapeutic concentrations due to high levels of plasma protein binding that limit the free ticagrelor available to bind to ENT-1.^14,28^ On the other hand, an effect of ticagrelor on adenosine uptake is more clearly seen at approximately therapeutic concentrations in the absence of plasma proteins.^29^ Since ticagrelor has been shown to induce a leftward shift in the dose-response curves for intravenous adenosine in studies of coronary blood flow responses and dyspnea severity, it remains likely that ticagrelor has an impact on the kinetics of adenosine uptake in vivo at the tissue level, such as in myocardium, that is not detected by the currently available blood assays, and more work is required to assess this.^13,30^

There were substantial numbers of patients with asymptomatic rises in troponin after PCI, but no evidence that ticagrelor was more effective than clopidogrel in attenuating troponin release, suggesting that the extent of myocardial injury induced by PCI is not usually sensitive to levels of platelet P2Y_12_ inhibition in a low-risk population. This observation is consistent with a previously reported small elective PCI study,^31^ but contrasts with another small study that demonstrated a reduced rate of MI with ticagrelor compared to clopidogrel.^32^ Larger clinical outcomes studies are in progress that will provide more definitive data on this comparison (NCT02617290 and NCT02548611).

This study was limited by a small sample size for assessing efficacy, safety, and tolerability, and a larger study is required to establish the benefits and risks of ticagrelor in stable CAD patients undergoing PCI. Our study simply provides pilot data for planning such a study. Only the impacts of ticagrelor on adenosine uptake in whole blood and circulating adenosine levels were assessed, not the impact of ticagrelor on tissue-level adenosine metabolism. The study was also not well powered for comparing the pharmacodynamic effects of the 2 maintenance doses of ticagrelor, although some significance was seen in predose levels of platelet reactivity suggesting that the 90 mg BID dose may have slightly greater consistency of effect than the 60 mg BID dose.

In conclusion, ticagrelor 60 mg and 90 mg BID regimens both achieved greater and more consistent platelet inhibition than standard clopidogrel therapy, but had no detectable impact on cellular adenosine uptake or circulating plasma adenosine concentration in stable CAD patients undergoing PCI. Further work is warranted to characterize the efficacy and safety of ticagrelor in this clinical setting.

## Appendix

Data Monitoring Committee Robert G. Wilcox (chair), University of Nottingham John T. Walsh, Nottingham University Hospitals NHS Trust William Smith, Nottingham University Hospitals NHS Trust Allan Skene, retired

## Acknowledgments

The authors are grateful to Drs Matthew Larman and Sven Nylander for their support. We also thank Drs Nana Theodorou and Erica Wallis, Clinical Research Office, Sheffield Teaching Hospitals National Health Service Foundation Trust, for their support.

## Sources of Funding

This study was funded by an investigator-initiated grant from AstraZeneca. The study was supported by the National Institute for Health Research award to the Sheffield National Institute for Health Research Clinical Research Facility. The views expressed are those of the authors and not necessarily those of the National Health Service, the National Institute for Health Research, or the Department of Health.

## Disclosures

Dr Storey reports institutional research grants/support from AstraZeneca and PlaqueTec; consultancy fees from AstraZeneca, Actelion, Avacta, Bayer, Bristol-Myers Squibb/Pfizer, Idorsia, Novartis, PlaqueTec, and The Medicines Company; and speaker fees from AstraZeneca. The other authors report no conflicts of interest.

## Supplementary Material
