# Supplementary material for: Study of Two Dose Regimens of Ticagrelor Compared With Clopidogrel in Patients Undergoing Percutaneous Coronary Intervention for Stable Coronary Artery Disease
Source: Circulation. 2018 Sep 24;138(13):1290–300. doi: 10.1161/CIRCULATIONAHA.118.034790 (PMC6159686; doi:10.1161/CIRCULATIONAHA.118.034790)
Supplement: Supplementary file 1 [file cir-138-1290-s001.pdf]

SUPPLEMENTAL MATERIAL

for

‘Study of Two Dose Regimens of Ticagrelor Compared with Clopidogrel in Patients Undergoing Percutaneous Coronary Intervention for Stable Coronary Artery Disease (STEEL-PCI)’

by Orme RC et al

Supplementary material for 'Study of Two Dose Regimens of Ticagrelor Compared with Clopidogrel in Patients Undergoing Percutaneous Coronary Intervention for Stable Coronary Artery Disease (STEEL-PCI)'

Influences of genetic variation on effects of clopidogrel and ticagrelor

Genetic variation in the activity of key cytochrome P450 (CYP) enzymes partly explains limited efficacy of clopidogrel in some individuals, including loss-of-function alleles in *CYP2C19* that have been associated with reduced clopidogrel active metabolite formation and increased risk of stent thrombosis.[1-5]

Genetic variants affecting ticagrelor and AR-C124910XX levels are uncommon and have limited effect on the levels as well as no detectable impact on efficacy or safety of ticagrelor.[6]

Inclusion and exclusion criteria for the study

Inclusion criteria

For inclusion in the study, subjects should fulfill the following criteria:

1. Provision of informed consent prior to any study specific procedures
2. Male or female aged greater than 18 years
3. Previous invasive coronary angiography with plan for PCI with coronary stent implantation for stable coronary artery disease

Exclusion criteria

Subjects should not enter the study if any of the following exclusion criteria are fulfilled:

1. Requirement for a chronic total occlusion to be crossed in order for *any* stent implantation to proceed
2. Plan for coronary angiography with a view to PCI if appropriate (i.e. current coronary anatomy not known)

3. Intention to use platelet function tests or genotyping to guide antiplatelet therapy
4. Known allergy to or intolerance of aspirin, clopidogrel or ticagrelor
5. Treatment with antiplatelet medication apart from aspirin or clopidogrel that cannot be stopped 10 days prior to PCI (e.g. ticagrelor, prasugrel, dipyridamole, ticlopidine, abciximab, tirofiban), for example because of continuing indication
6. Planned treatment or consideration of treatment with oral antiplatelet medication other than aspirin or clopidogrel following PCI
7. Planned use of a glycoprotein IIb/IIIa antagonist for the PCI procedure
8. Myocardial infarction within the past 12 months
9. Current or planned use of an oral anticoagulant (e.g. warfarin, dabigatran, rivaroxaban, apixaban)
10. Previous history of intracranial haemorrhage or other intracranial pathology associated with increased bleeding risk
11. Haemoglobin < 100 g/L or other evidence of active bleeding
12. Peptic ulceration documented by endoscopy within the last 3 months unless healing proven by repeat endoscopy
13. History of acute or chronic liver disease (e.g. cirrhosis)
14. Treatment in the last 10 days or requirement for ongoing treatment with a strong CYP3A4 inhibitor (e.g. ketoconazole, itraconazole, voriconazole, telithromycin,clarithromycin, nefazadone, ritonavir, saquinavir, nelfinavir, indinavir, atanazavir, or over 1 litre daily of grapefruit juice) or inducer (e.g. rifampin/rifampicin, rifabutin, phenytoin, carbamazepine, phenobarbital)
15. Requirement for ongoing treatment with simvastatin or lovastatin at a dose greater than 40 mg per day

16. Treatment with a CYP3A4 substrate with a narrow therapeutic index (e.g. cyclosporine, quinidine)
17. Requirement for ongoing treatment with a moderate-or-strong CYP2C19 inhibitor that is known to or predicted to impair the response to clopidogrel (omeprazole, esomeprazole, fluconazole, fluvoxamine, fluoxetine, moclobemide, voriconazole ciprofloxacin, cimetidine, carbamazepine, oxcarbazepine, or chloramphenicol)
18. End-stage renal failure requiring dialysis
19. History of alcohol or drug abuse in the last year
20. Co-morbidity associated with life expectancy less than 1 year
21. Females of child-bearing potential unless negative pregnancy test at screening and willing to use effective contraception (i.e. established use of oral, injected or implanted hormonal methods of contraception *or* placement of an intrauterine device (IUD) or intrauterine system (IUS) *or* barrier methods of contraception with spermicide *or* sole male partner with prior vasectomy and confirmed absence of sperm in ejaculate) for the duration of treatment with study medication
22. Any other condition deemed by the investigator to place the patient at excessive risk of bleeding with ticagrelor

High-performance liquid chromatography (HPLC) methods

Samples with stop solution were centrifuged at 1500*g* and the supernatant deproteinised by the addition of ice-cold 70% perchloric acid before further centrifugation at 13000*g* and storage of the supernatant at -80°C. HPLC was performed using a Waters 2695 HPLC analyser and a Waters 2487 Ultra-Violet/Visible (UV/Vis) detector with wavelength 258 nm. Data was collected by DataApex Clarity software. The column was a Waters Xbridge C18 5um (250mm x 4.6mm id) at a

temperature of 23°C. The mobile phase was 2% acetonitrile in aqueous ammonium hydroxide (0.1%) for 35 mins ramping to 98% acetonitrile after 36 mins until 40 mins (1mL/min). The injection volume was 100uL. The lower limit of quantification for adenosine was 0.01 μmol/L.

Genetic analysis methods

DNA was extracted from whole blood samples using Chemagen Chemagic 10k kits (Perkin Elmer, Baesweiler, Germany) followed by elution in Tris-EDTA buffer. The DNA was quantified using Quantifluor® dsDNA System (Promega, Madison, WI, USA). Genotyping was done using Taqman assays (Applied Biosystems, Life Technologies, Pleasanton, CA, USA) using an Applied Biosystems 7900HT Real-Time PCR System. The alleles genotyped included: *CYP2C19* loss-of-function alleles *2 (rs4244285), *3 (rs4986893), *4 (rs28399504), *5 (rs56337013), *6 (rs72552267) *7 (rs72558186) and *8 (rs41291556); *CYP2C19* gain-of-function allele *17 (rs12248560); *CYP3A43* (rs62471956); *UGT2B7* (glucuronosyl transferase family 2 member B7) (rs61361928); and *SLC01B1* (solute carrier organic anion transporter family member 1B1 (rs4149056).

Excluded patient on strong CYP3A inducer

One patient randomized to ticagrelor 60mg bid was subsequently found to have been taking a strong CYP3A inducer throughout the study and was, therefore, included in error. Their pharmacodynamic and pharmacokinetic data were excluded from the main analyses to avoid misleading comparison of the groups. It was confirmed that no other patients in the study received excluded medication. The patient was informed of this error and agreed for their individual data to be presented anonymously in view of the scientific interest. After ticagrelor 180-mg loading dose, their VerifyNow P2Y12 assay

showed PRU 220 and percentage inhibition 21%. Pre- and post-maintenance dose of ticagrelor 60mg at 1 month, these values were 223 and 4% pre-dose and 208 and 21% post-dose, respectively. LTA results were consistent with these values. Corresponding to these low levels of platelet $P2Y_{12}$ inhibition, plasma levels of ticagrelor and AR-C124910XX were also low, indicating ultra-rapid metabolism of ticagrelor and its active metabolite: following ticagrelor 180-mg loading dose, levels were 55 and 105 ng/mL, respectively; pre-maintenance dose, levels were 7.5 and 36.4 ng/mL, respectively, and post-maintenance dose, levels were 18.6 and 60 ng/mL, respectively.

These findings illustrate the importance of checking on relevant CYP3A-mediated drug interactions when using ticagrelor and avoiding the use of ticagrelor in patients receiving strong CYP3A inducers.

Supplementary genetic analyses

The presence of a gain-of-function allele for CYP2C19 did not influence the relationship between clopidogrel and either of the ticagrelor doses (Supplementary Table 4 and 5).

None of the patients carried the rare alleles for *CYP3A43* (rs62471956) or *UGT2B7* (rs61361928). There were no consistent effects of the variant of *SLC01B1* (rs4149056) on platelet reactivity or plasma ticagrelor levels (Supplementary Tables 6 to 9).

Supplementary Table 1. Characteristics of all randomised patients

	Clopidogrel<br>n=60	Ticagrelor 60mg<br>n=60	Ticagrelor 90mg<br>n=60
Age, years, mean (SD)	63.7 (11.9)	67 (8.6)	64.9 (8.3)
Male sex, n (%)	47 (78.3%)	50 (85.2%)	50 (85.2%)
Body weight, kgs, median (interquartile range)	85.5 (77-102)	87.5 (73-96)	85 (79-98)
Body mass index, mean (SD)	30.3 (5.7)	28.8 (3.7)	30 (4.9)
Race, n (%)			
White	59 (98.3%)	59 (98.3%)	58 (96.7%)
Black	1 (1.7%)	0 (0%)	1 (1.7%)
Asian	0 (0%)	1 (1.7%)	1 (1.7%)
Cardiovascular risk factors, n (%)			
Current smoker	7 (11.7%)	3 (5%)	7 (11.7%)
Hypertension	42 (70%)	37 (61.7%)	41 (68.3%)
Dyslipidemia	54 (90%)	54 (90%)	58 (96.6%)
Diabetes mellitus	13 (21.7%)	8 (13.3%)	13 (21.7%)
Medical history, n (%)			
Myocardial infarction	9 (15%)	7 (11.7%)	4 (6.7%)
PCI	6 (10%)	7 (11.7%)	8 (13.3%)
CABG	3 (5%)	5 (8.3%)	3 (5%)
Cardiac failure	5 (8.3%)	2 (3.3%)	2 (3.3%)
Transient ischemic attack	3 (5%)	3 (5%)	3 (5%)
Non-hemorrhagic stroke	1 (1.7%)	0 (0%)	2 (3.3%)
Peripheral arterial disease	7 (11.6%)	5 (8.3%)	3 (5%)
COPD	6 (10%)	6 (10%)	3 (5%)
Concomitant medication, n (%)			
Aspirin 75mg daily	60 (100%)	60 (100%)	60 (100%)
Beta-blocker	53 (88.3%)	45 (75%)	41 (68.3%)*
ACE inhibitor	16 (26.7%)	19 (31.7%)	18 (30%)
Statin	54 (90%)	54 (90%)	52 (86.7%)

*All comparisons between the groups are not significant other than treatment with beta-blocker (p=0.03). SD: standard deviation.

Supplementary Table 2. Baseline demographic and procedural characteristics and medications at 1 month

	Clopidogrel<br>n=53	Ticagrelor 60mg<br>n=53	Ticagrelor 90mg<br>n=48
Age, years, mean (SD)	65.0 (8.4)	66.6(8.4)	66(7.747)
Male sex, n (%)	43 (81.1%)	45 (84.9%)	40 (83.3%)
Body weight, kgs, median (IQR)	87.0 (77-102)	68.2(60.6-72.2)	85(79-98)
Body mass index, mean (SD)	30.51(5.75)	28.85(3.7)	30.07 (4.738)
Race, n (%)			
White	52 (98.1%)	52 (98.1%)	46 (95.8%)
Black	1 (1.9%)	0 (0%)	1 (2.1%)
Asian	0 (0%)	1 (1.9%)	1 (2.1%)
Cardiovascular risk factors, n (%)			
Current smoker	6 (11.3%)	2 (3.8%)	4 (8.3%)
Hypertension	37 (69.8%)	31 (58.5%)	31 (64.6%)
Dyslipidemia	37 (69.8%)	47 (88.7%)	46 (95.8%)
Diabetes mellitus	11 (20.8%)	7 (13.2%)	11 (22.9%)
Medical history, n (%)			
Myocardial infarction	8 (15.1%)	7 (13.2%)	4 (8.3%)
PCI	5 (9.4%)	7 (13.2%)	7 (14.6%)
CABG	3 (5.7%)	4 (7.5%)	1 (1.9%)
Cardiac failure	5 (9.4%)	1 (1.9%)	0 (0%)
Transient ischemic attack	3 (5.7%)	3 (5.7%)	2 (3.8%)
Non-hemorrhagic stroke	1 (1.9%)	0 (0%)	1 (1.9%)
Peripheral arterial disease	5 (9.4%)	3 (5.7%)	3 (6.25%)
COPD	4 (7.6%)	1 (1.9%)	2 (4.2%)
Concomitant medication, n (%)			
Aspirin 75mg daily	53 (100%)	53 (100%)	47 (97.9%)
Beta-blocker	46 (86.8%)	40 (75.5%)	31 (64.6%)
ACE inhibitor	15 (28.3%)	18 (34.0%)	11 (22.9%)
Statin	47 (88.7%)	47 (88.7%)	41 (85.4%)
CYP2C19 LOF carrier, n (%)	17 (32.1%)	18 (34.0%)	9 (18.8%)
Procedural characteristics			
Number of vessels treated, mean (SD)	1.2 (0.45)	1.23 (0.42)	1.25 (0.48)
Number of lesions treated, mean (SD)	1.51 (0.78)	1.53 (0.7)	1.44 (0.68)
Total stent length, mm, mean (SD)	37.5 (25.4)	39.2 (23.4)	36.7 (24.4)
Minimum stent diameter, mm, mean (SD)	3.03 (0.53)	2.98 (0.49)	3.03 (0.48)
Bifurcation treated, n (%)	1 (1.9%)	4 (7.5%)	2 (4.2%)
Left main stem treated, n (%)	1 (1.9%)	3 (5.7%)	2 (4.2%)
Chronic total occlusion treated, n (%)			
Arterial Access, n (%)			

Radial	41 (77.4%)	40 (75.5%)	35 (72.9%)
Femoral	10 (18.9%)	13 (24.5%)	12 (25%)
Radial-to-femoral	2 (3.8%)	0 (0%)	1 (2.1%)
Brachial	0 (0%)	0 (0%)	0 (0%)

SD: standard deviation. PCI: percutaneous coronary intervention. CABG: coronary artery bypass graft surgery. COPD: chronic obstructive pulmonary disease. ACE: angiotensin-converting enzyme. CYP2C19 LOF: loss-of-function allele carrier for cytochrome P450 2C19.

Supplementary Table 3. Adverse events

	Clopidogrel N= 60	Ticagrelor 60mg N = 56	Ticagrelor 90mg N = 58
**Serious Adverse Events**	N (%)	N (%)	N (%)
**Procedural**			
Arterial access site bleeding	2 (3.3)	2 (3.6)	0 (0)
Arterial access site haematoma	0 (0)	1 (1.8)	2 (4)
Pericardial effusion	0 (0)	1 (1.8)	0 (0)
Radial artery dissection	0 (0)	0 (0)	1 (1.7)
**Non-procedural**			
Chest pain	2 (3.3)	2 (3.6)	0 (0)
Palpitations	0 (0)	0 (0)	1 (1.7)
Vasovagal syncope	0 (0)	1 (1.8)	1 (1.7)
Systemic thromboembolism	0 (0)	0 (0)	1 (1.7)
Venous thromboembolism	1 (1.7)	0 (0)	0 (0)
**Adverse Events**	N (%)	N (%)	N (%)
**Procedural**			
Coronary artery haematoma	0 (0)	1 (1.8)	0 (0)
**Non-procedural**			
Hypertension	0 (0)	0 (0)	1 (1.7)
Palpitations	0 (0)	1 (1.8)	0 (0)
Oedema	0 (0)	1 (1.8)	0 (0)
Pre-syncope or syncope	0 (0)	2 (3.6)	1 (2)
Dyspnoea	0 (0)	4 (7.1)	11 (19.0)**
Anaemia	0 (0)	1 (1.8)	0 (0)
Bruising	2 (3.3)	1 (1.8)	1 (1.7)
Epistaxis	0 (0)	0 (0)	1 (1.7)
Fatigue	0 (0)	2 (3.6)	0 (0)
Gastrointestinal symptoms	3 (5.0)	3 (5.4)	2 (3.4)
Gout	1 (1.7)	1 (1.8)	0 (0)
Haematospermia/haematuria	0 (0)	0 (0)	2 (3.4)
Non-cardiac chest pain	2 (3.3)	3 (5.4)	6 (10.3)
Rash	0 (0)	1 (1.8)	1 (1.7)
Shingles	1 (1.7)	0 (0)	1 (1.7)

Group comparisons performed using Fisher's exact test: ** P < 0.001 vs clopidogrel. All other P > 0.05.

Supplementary Table 4. VerifyNow $P2Y_{12}$ results following standard loading regimens of clopidogrel or ticagrelor at the time of PCI according to CYP2C19 loss-of-function allele carrier status

***CYP2C19* genotype status**	**Clopidogrel**		**Ticagrelor**		**P value**
	**n**	**Mean ± SD**	**n**	**Mean ± SD**	
**LOF**					
% inhibition	18	17 ± 24	31	83 ± 24	<0.001
PRU	18	200 ± 72	31	35 ± 49	<0.001
**No LOF**					
% inhibition	41	32 ± 25	79	87 ± 16	<0.001
PRU	41	166 ± 66	79	30 ± 33	<0.001

LOF: *CYP2C19* loss-of-function allele carrier. PRU: $P2Y_{12}$ reaction units.

Supplementary Table 5. VerifyNow $P2Y_{12}$ results following one month of clopidogrel or ticagrelor according to CYP2C19 carrier status

VerifyNow P2Y12 assay	Clopidogrel		Ticagrelor 60mg		Ticagrelor 90mg		P value Clop vs T60mg	P value Clop vs T90mg	P value T60mg vs T90mg
	n	Mean ± SD	n	Mean ± SD	n	Mean ± SD			
**LOF**									
PRU Pre dose	16	187 ± 47	16	77 ± 67	8	22 ±18	0.0003	0.0001	0.023
PRU Post dose	16	176 ± 60	16	35 ± 25	8	8 ± 3	<0.0001	<0.0001	0.003
% inhibition Pre dose	16	19 ± 19	16	67 ± 27	8	90 ± 9	0.0002	<0.0001	0.038
% inhibition Post dose	16	22 ± 24	16	86 ± 10	8	97 ±1	<0.0001	<0.0001	0.003
**No LOF**									
PRU Pre dose	34	178 ± 43	34	55 ± 34	36	42 ± 39	<0.0001	<0.0001	0.11
PRU Post dose	36	155 ± 49	35	32 ± 32	39	27 ± 22	<0.0001	<0.0001	0.64
% inhibition Pre dose	34	22 ± 17	34	75 ± 15	36	82 ± 17	<0.0001	<0.0001	0.03
% inhibition Post dose	36	37 ± 20	35	86 ± 15	39	89 ± 10	<0.0001	<0.0001	0.56

LOF: CYP2C19 loss-of-function allele carrier. PRU: $P2Y_{12}$ reaction units.

Supplementary Table 6. VerifyNow $P2Y_{12}$ results following standard loading regimens of clopidogrel or ticagrelor at the time of PCI according to CYP2C19 gain of function allele carrier status

VerifyNow results according to CYP2C19 genotype	Clopidogrel		Ticagrelor		p value
	n	Mean ± SD	n	Mean ± SD	
**GOF**					
% inhibition	18	30 ± 23	34	86 ± 19	<0.0001
PRU	18	164 ± 60	34	29 ± 36	<0.0001
**No GOF**					
% inhibition	41	26 ± 26	76	85 ± 19	<0.0001
PRU	41	181 ± 73	76	33 ± 39	<0.0001

Supplementary Table 7. VerifyNow P2Y12 assay results following one month of clopidogrel or ticagrelor according to *CYP2C19* gain-of-function carrier status

VerifyNow P2Y12 assay	Clopidogrel		Ticagrelor 60mg		Ticagrelor 90mg		P value Clop vs T60mg	P value Clop vs T90mg	P value T60mg vs T90mg
	n	Mean ± SD	n	Mean ± SD	n	Mean ± SD			
**GOF**									
PRU Pre dose	15	170 ± 50	12	61 ± 39	16	47 ± 47	<0.0001	<0.0001	0.3067
PRU Post dose	16	148 ± 58	12	35 ± 41	17	28 ± 26	<0.0001	<0.0001	0.7027
% inhibition Pre dose	15	19 ± 19	12	67 ± 27	16	90 ± 9	<0.0001	<0.0001	0.4419
% inhibition Post dose	16	37 ± 23	12	85 ± 19	17	88 ± 11	<0.0001	<0.0001	0.8525
**No GOF**									
PRU Pre dose	35	185 ± 41	38	62 ± 51	28	34 ± 29	<0.0001	<0.0001	0.0119
PRU Post dose	37	163 ± 57	39	33 ± 26	30	21 ± 19	<0.0001	<0.0001	0.0659
% inhibition Pre dose	35	21 ± 17	38	73 ± 21	28	85 ± 13	<0.0001	<0.0001	0.0026
% inhibition Post dose	37	30 ± 22	39	86 ± 11	30	91 ± 18	<0.0001	<0.0001	0.04

Supplementary Table 8. VerifyNow P2Y12 assay results following standard loading regimens of clopidogrel or ticagrelor at the time of PCI according to SLC01B1 genotype carrier status

VerifyNow results according to SLC01B1 genotype	Clopidogrel		Ticagrelor		p value
	n	Mean ± SD	n	Mean ± SD	
**Carrier**					
% inhibition	15	22 ± 22	25	86 ± 20	<0.0001
PRU	15	191 ± 53	25	37 ± 56	<0.0001
**Non-carrier**					
% inhibition	44	29 ± 26	84	96 ± 16	<0.0001
PRU	44	171 ± 74	84	30 ± 31	<0.0001

Supplementary Table 9. VerifyNow P2Y12 assay results following one month of clopidogrel or ticagrelor according to SLC01B1 genotype carrier status

SLC01B1 genotype	Clopidogrel		Ticagrelor 60mg		Ticagrelor 90mg		P value Clop vs T60mg	P Value Clop vs T90mg	P value T60mg vs T9
	n	Mean ± SD	n	Mean ± SD	n	Mean ± SD			
**Carrier**									
PRU Pre dose	14	193 ± 31	14	39 ± 21	10	45 ± 54	<0.0001	<0.0001	0.594
PRU Post dose	10	172 ± 37	14	23 ± 19	10	22 ± 23	<0.0001	<0.0001	0.965
% inhibition Pre dose	14	20 ± 17	14	83 ± 9	10	79 ± 26	<0.0001	<0.0001	0.635
% inhibition Post dose	10	32 ± 18	14	91 ± 8	10	90 ± 10	<0.0001	<0.0001	0.784
**Non-carrier**									
PRU Pre dose	36	176 ± 48	36	71 ± 52	34	37 ± 30	<0.0001	<0.0001	0.0023
PRU Post dose	39	159 ± 58	37	37 ± 32	37	24 ± 22	<0.0001	<0.0001	0.0622
% inhibition Pre dose	36	22 ± 18	36	69 ± 22	34	85 ± 13	<0.0001	<0.0001	0.0006
% inhibition Post dose	39	33 ± 24	37	84 ± 15	37	90 ± 9	<0.0001	<0.0001	0.0681

Supplementary Table 10. Plasma ticagrelor and AR-C124910XX results at time of PCI according to SLC01B1 carrier status

	Carrier		Non-carrier		P
	n	Mean ± SD	n	Mean ± SD	value
**Ticagrelor (ng/mL)**	26	1126 ± 658	84	1097 ± 516	0.976
**AR-C124910XX (ng/mL)**	26	223 ± 153	84	218 ± 117	0.687

Supplementary Table 11. Plasma ticagrelor and AR-C124910XX results at one month according to SLC01B1 carrier status

	Carrier		Non-carrier		P
	n	Mean ± SD	n	Mean ± SD	value
**Ticagrelor (ng/mL)**					
T60 pre dose	14	277 ± 152	36	280 ± 242	0.627
T60 post dose	14	521 ± 318	34	498 ± 272	0.912
T90 pre dose	10	342 ± 204	37	375 ± 187	0.626
T90 post dose	10	813 ± 433	37	764 ± 332	0.715
**AR-C124910XX (ng/mL)**					
T60 pre dose	14	94 ± 47	36	95 ± 60	0.737
T60 post dose	14	129 ± 59	34	130 ± 80	0.665
T90 pre dose	10	126 ± 51	37	132 ± 73	0.576
T90 post dose	10	208 ± 84	37	198 ± 101	0.461

T60: Ticagrelor 60mg group; T90: Ticagrelor 90mg group

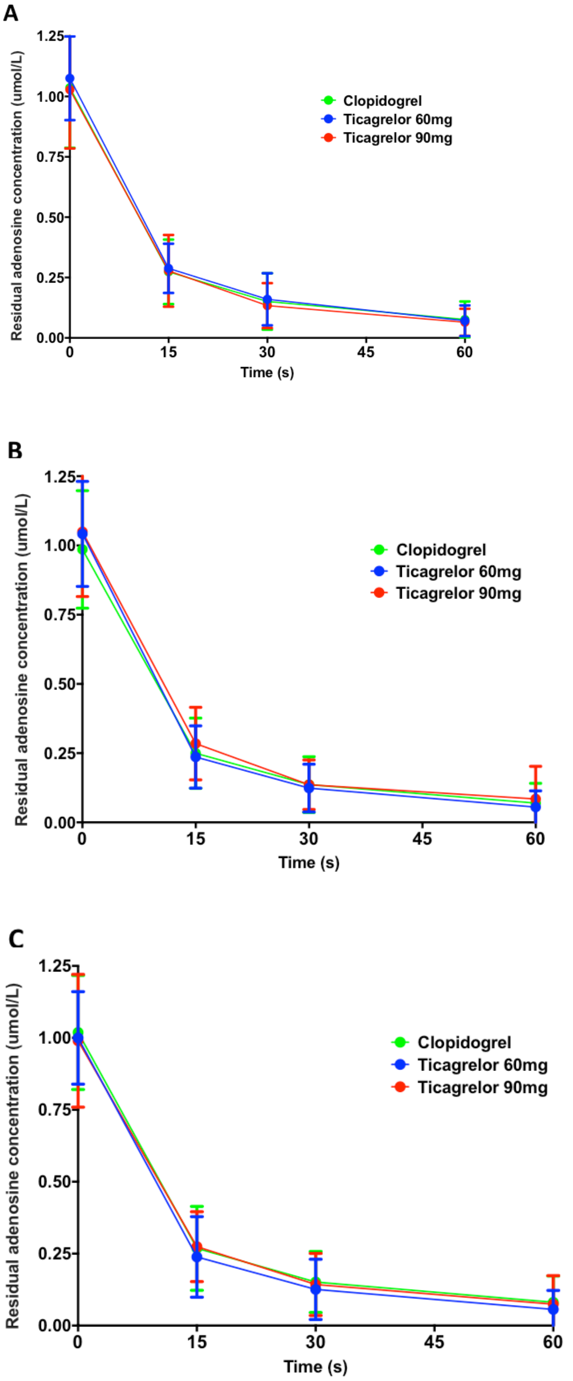

Supplementary Figure 1. Time course of whole blood adenosine uptake

Residual adenosine levels at 0 to 60 seconds after mixing adenosine 1 μmol/L with blood samples obtained (A) at the time of PCI following a standard loading regimen of clopidogrel (n=54) or 180-mg loading dose of ticagrelor (n=50 and 54 for 60mg and 90mg groups, respectively); and after one month of treatment (B) pre-maintenance dose and (C) post-maintenance dose for each of the three treatment groups (clopidogrel 75mg qd: n=45; ticagrelor 60mg bid: n=46; and ticagrelor 90mg bid; n=43 & 45). Data are mean ± SD.

Supplementary Figure 2A. Post loading dose

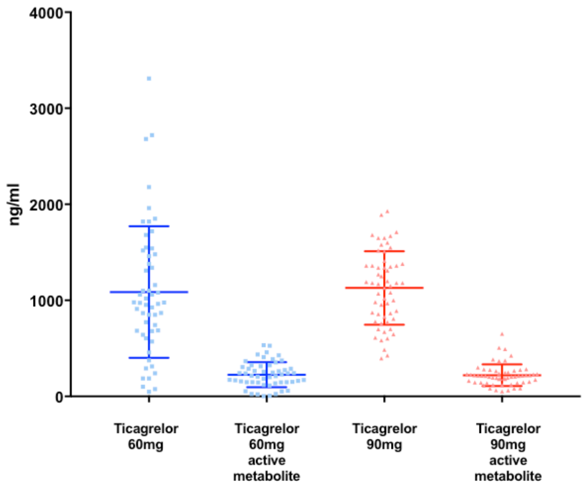

Supplementary Figure 2B. At one month

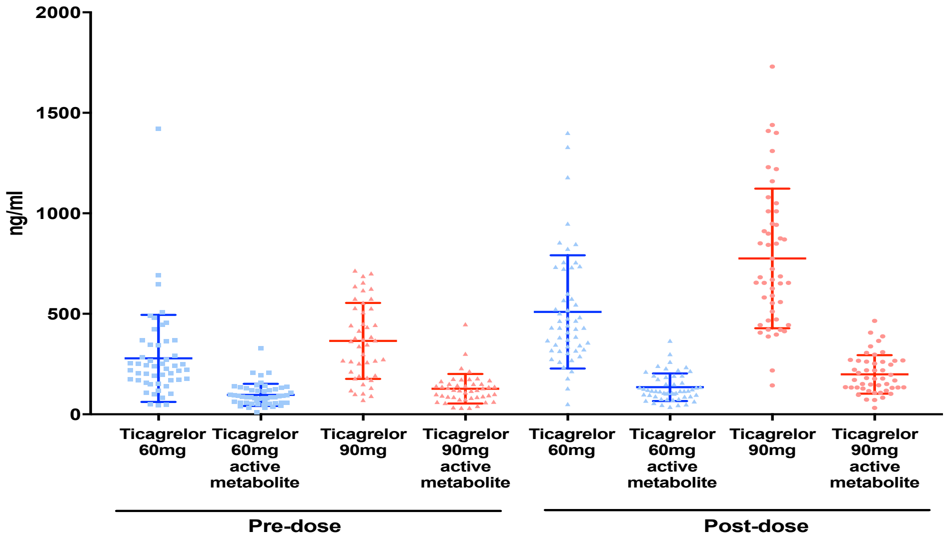

Supplementary Figure 2. Ticagrelor and Active Metabolite AR-C124910XX Plasma Concentration

Individual results for plasma concentrations of ticagrelor and active metabolite AR-C124910XX (A) following a standard loading dose of ticagrelor and (B) after one month, pre-maintenance dose and post-maintenance dose with either ticagrelor 60 mg or ticagrelor 90 mg twice daily. Solid lines with error bars indicate mean ± SD.

References

1. Price MJ, Murray SS, Angiolillo DJ, Lillie E, Smith EN, Tisch RL, Schork NJ, Teirstein PS and Topol EJ. Influence of Genetic Polymorphisms on the Effect of High- and Standard-Dose Clopidogrel After Percutaneous Coronary Intervention: The GIFT (Genotype Information and Functional Testing) Study. *J Am Coll Cardiol*. 2012;59:1928-1937.
2. Tantry US, Bliden KP, Wei C, Storey RF, Armstrong M, Butler K and Gurbel PA. First Analysis of the Relation Between CYP2C19 Genotype and Pharmacodynamics in Patients Treated With Ticagrelor Versus Clopidogrel: The ONSET/OFFSET and RESPOND Genotype Studies. *Circulation Cardiovascular Genetics*. 2010;3:556-66.
3. Wallentin L, James S, Storey RF, Armstrong M, Barratt BJ, Horrow J, Husted S, Katus H, Steg PG, Shah SH and Becker RC. Effect of CYP2C19 and ABCB1 single nucleotide polymorphisms on outcomes of treatment with ticagrelor versus clopidogrel for acute coronary syndromes: a genetic substudy of the PLATO trial. *Lancet*. 2010;376:1320-8.
4. Aradi D, Storey RF, Komócsi A, Trenk D, Gulba D, Kiss RG, Husted S, Bonello L, Sibbing D, Collet J-P and Huber K. Expert position paper on the role of platelet function testing in patients undergoing percutaneous coronary intervention. *Eur Heart J*. 2014;35:209-15.
5. Bonello L, Tantry US, Marcucci R, Blindt R, Angiolillo DJ, Becker R, Bhatt DL, Cattaneo M, Collet JP, Cuisset T, Gachet C, Montalescot G, Jennings LK, Kereiakes D, Sibbing D, Trenk D, Van Werkum JW, Paganelli F, Price MJ, Waksman R and Gurbel PA. Consensus and Future Directions on the Definition of High On-Treatment Platelet Reactivity to Adenosine Diphosphate. *J Am Coll Cardiol*. 2010;56:919-933.
6. Varenhorst C, Eriksson N, Johansson Å, Barratt BJ, Hagström E, Åkerblom A, Syvänen AC, Becker RC, James SK, Katus HA, Husted S, Steg PG, Siegbahn A, Voora D, Teng R, Storey RF and Wallentin L. Effect of genetic variations on ticagrelor plasma levels and clinical outcomes. *Eur Heart J*. 2015;36:1901-12.
